# Regulation of Ras homolog family member G by microRNA-124 regulates proliferation and migration of human retinal pigment epithelial cells

**DOI:** 10.1038/s41598-020-72360-5

**Published:** 2020-09-22

**Authors:** Jong Hwa Jun, Myeong-Jin Son, Hyun-Gyo Lee, Kyu Young Shim, Won-Ki Baek, Jae-Young Kim, Choun-Ki Joo

**Affiliations:** 1 Department of Ophthalmology, Dongsan Medical Center, Keimyung University School of Medicine, Daegu, Korea; 2 Department of Microbiology, Keimyung University School of Medicine, Daegu, Korea; 3 Department of Oral Biochemistry, School of Dentistry, IHBR, Kyungpook National University, Daegu, Korea; 4 Department of Ophthalmology and Visual Science, Seoul St. Mary’s Hospital, College of Medicine, The Catholic University of Korea, Seoul, Korea

**Keywords:** Molecular biology, Molecular medicine

## Abstract

Uncontrolled retinal pigment epithelial (RPE) cell proliferation/migration contribute to the pathological tractional membrane development in proliferative vitreoretinopathy. Recent studies reported that microRNA (miR)-124 controls various cellular functions via the direct targeting of small Ras homolog family member G (RHOG). Therefore, we investigated the role of the neuron-specific miR-124 and RHOG in RPE cell proliferation/migration. Alterations in miR-124 and RhoG expression, as per cell confluence were evaluated through quantitative real-time PCR and western blotting, respectively. After transfection with miR-124, we quantified RPE cell viability and migration and observed cell polarization and lamellipodia protrusions. We evaluated the expression of RHOG/RAC1 pathway molecules in miR-124-transfected RPE cells. Endogenous miR-124 expression increased proportionally to RPE cell density, but decreased after 100% confluence. Overexpression of miR-124 decreased cell viability and migration, BrdU incorporation, and Ki-67 expression. Inhibition of endogenous miR-124 expression promoted RPE cell migration. Transfection with miR-124 reduced cell polarization, lamellipodia protrusion, and *RHOG* mRNA 3′ untranslated region luciferase activity. Like miR-124 overexpression, RhoG knockdown decreased RPE cell viability, wound healing, and migration, and altered the expression of cell cycle regulators. These results suggest that miR-124 could be a therapeutic target to alleviate fibrovascular proliferation in retinal diseases by regulating RPE proliferation/migration via RHOG.

## Introduction

Members of the small GTPase Ras homolog (RHO) family serve as molecular switches for complex cellular processes, such as migration, division, and cell cycle progression, although they are primarily known as actin cytoskeleton regulators^1^. Phylogenetic analysis revealed that the RHO family comprises two exclusive groups; the first group includes RHOA, B, and C, and the second includes RHOG and RAC1/2^2^. Vincent et al. first described Ras homolog growth-related (RHOG) based on its growth-related functions^2^; however, the underlying functions and regulatory mechanisms of RhoG protein are largely unknown. Recently, a subset of small non-coding RNAs, microRNAs (miRNAs), were identified as endogenous regulatory RNA molecules; they play pivotal roles in the regulation of ~ 30% of all mammalian genes, mainly via repression^3,4^.

miR-124 is abundant in the brain and accounts for 25–48% of all central nervous system (CNS)-expressed miRNAs^5,6^. From the developmental perspective, the majority of CNS-specific cells begin in the neural ectoderm, including retinal pigment epithelial (RPE) cells. We hypothesized that miR-124 would be expressed in the neural retina, as it was previously identified in the murine eye by miRNA profiling^7,8^. The retinal pigment epithelium physiology presents unique challenges that necessitate precisely tuned regulatory networks to maintain cell viability. In steady-state conditions, RPE cells remain in the quiescent stage of the cell cycle^9,10^. Under pathological conditions, RPE cells are vulnerable to insults caused by the aging process and environmental stressors; proliferative vitreoretinopathy^11,12^, diabetic retinopathy^13^, and idiopathic epiretinal membranes^14^ can be induced by uncontrolled proliferation and RPE cell migration into vitreous space. Emerging evidence suggests that fine-tuning by miRNAs is essential for responses to cellular stress^15^; miRNAs play a critical role in the survival and physiology of photoreceptor cells and the retinal pigment epithelium, making them putative therapeutic targets in these blinding diseases. Furthermore, Franke et al.^16^ confirmed that miR-124 regulates neuronal differentiation by targeting RHOG.

In the present study, we investigated the regulatory functions of miR-124 in RPE cells; we focused particularly at cell proliferation and migration. Additionally, we assessed the physiological alterations caused by *RHOG* gene silencing in RPE cells, using small interfering RNAs (siRNAs).

## Results

### Endogenous expression levels of miR-124 and RhoG inversely correlated with RPE cell confluence

**Figure 1:**
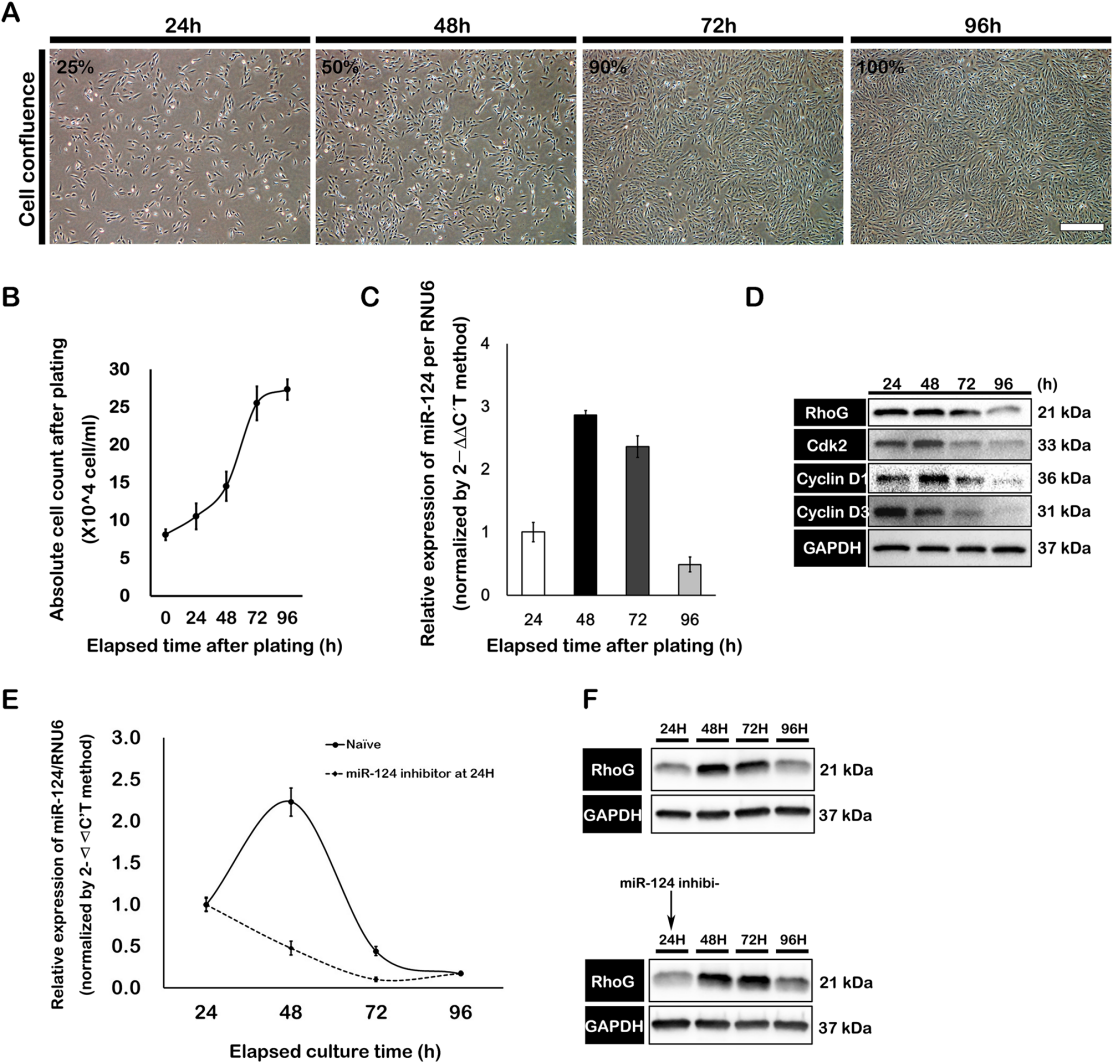
Analysis of miR-124 and RhoG expression during cell proliferation. (**A**) Representative images of cell confluence after plating 7.5 × 10^4^ RPE cells/mL in 6-well cell culture plates. (**B**) At the indicated time points, RPE cells were counted in triplicate cell culture wells. (**C**) Quantitative measurements of endogenous miR-124 expression levels in RPE cells at the indicated time points. The quantitative miRNA expression results were normalized to *RNU6* and miR-16 expression levels. (**D**) The protein levels of cell cycle regulatory factors and RhoG were analyzed by western blotting. (**E**) Quantitative evaluation of endogenous miR-124 expression in non-treated or miR-124 inhibitor-treated RPE cells. (**F**) Expression of RhoG in non-treated or miR-124 inhibitor-treated RPE cells was assessed using western blotting.

RPE cells (7.5 × 10^4^ cells/mL) reached confluences of approximately 25%, 50%, 90%, and 100% after 24, 48, 72, and 96 h, respectively (Fig. 1A, B). The endogenous expression of miR-124 rapidly increased to threefold at 48 h compared to the level at 24 h and was maintained until 72 h after plating. However, its expression abruptly decreased at 96 h, to less than half the initial expression level, when RPE cells reached full confluence (Fig. 1C). In addition, RhoG protein expression decreased up to 72 h after plating; of note, it significantly decreased below the levels at 24, 48, and 72 h when the cells reached 100% confluence (Fig. 1D). To inhibit endogenously expressed miR-124, a miR-124-specific inhibitor was used, 24 h after RPE cell plating. As expected, the intracellular miR-124 expression levels continuously decreased up to 72 h (Fig. 1E). On the other hand, RhoG expression was increased compared to that in non-treated cells from 48 to 72 h, as per the western blot analysis (Fig. 1F).

### Overexpression of miR-124 reduced RPE cell numbers and proliferative ability

**Figure 2:**
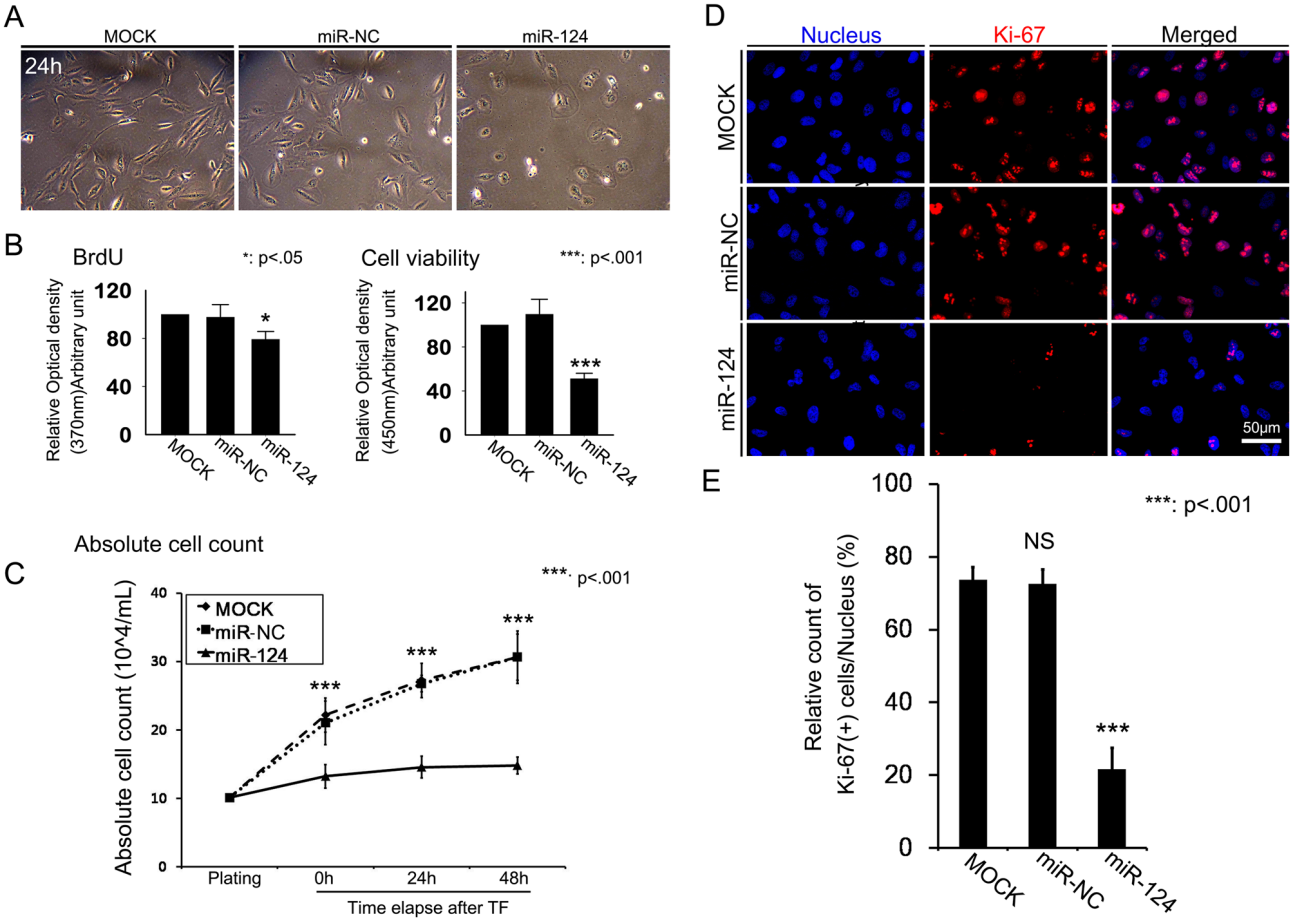
Effects of miR-124 transfection on RPE cell proliferation. (**A**) Representative images of RPE cells after transfection with miR-124. (**B**) Quantitative measurement of proliferation via BrdU incorporation and viability via WST-8 assays. (**C**) Absolute cell counts after transfection with miR-124. (**D**) Ki-67 immunostaining after transfection with miR-124. (**E**) Percentage of Ki-67-positive cells of all cells with DAPI-positive nuclear staining. Data represent mean ± standard deviation (SD). Data analyzed by one way-ANOVA followed by Turkey’s HSD test. *p < 0.05; ***p < 0.001.

After we transfected RPE cells with miR-124, we evaluated cell density through phase-contrast microscopy. The introduction of miR-124 decreased cell density, as compared with miR-NC transfection, at 24 h (Fig. 2A). We also found that miR-124 overexpression decreased cell proliferation and viability (Fig. 2B). In addition, using an absolute cell counting method, miR-124 transfection decreased cell counts by approximately 3 folds (Fig. 2C). Overexpression of miR-124 significantly decreased the number of Ki-67-positive cells within the DAPI-positive cells, as compared with mock control or miR-NC introduction (Fig. 2D, E).

### Overexpression of miR-124 impeded the motility of RPE cells

**Figure 3:**
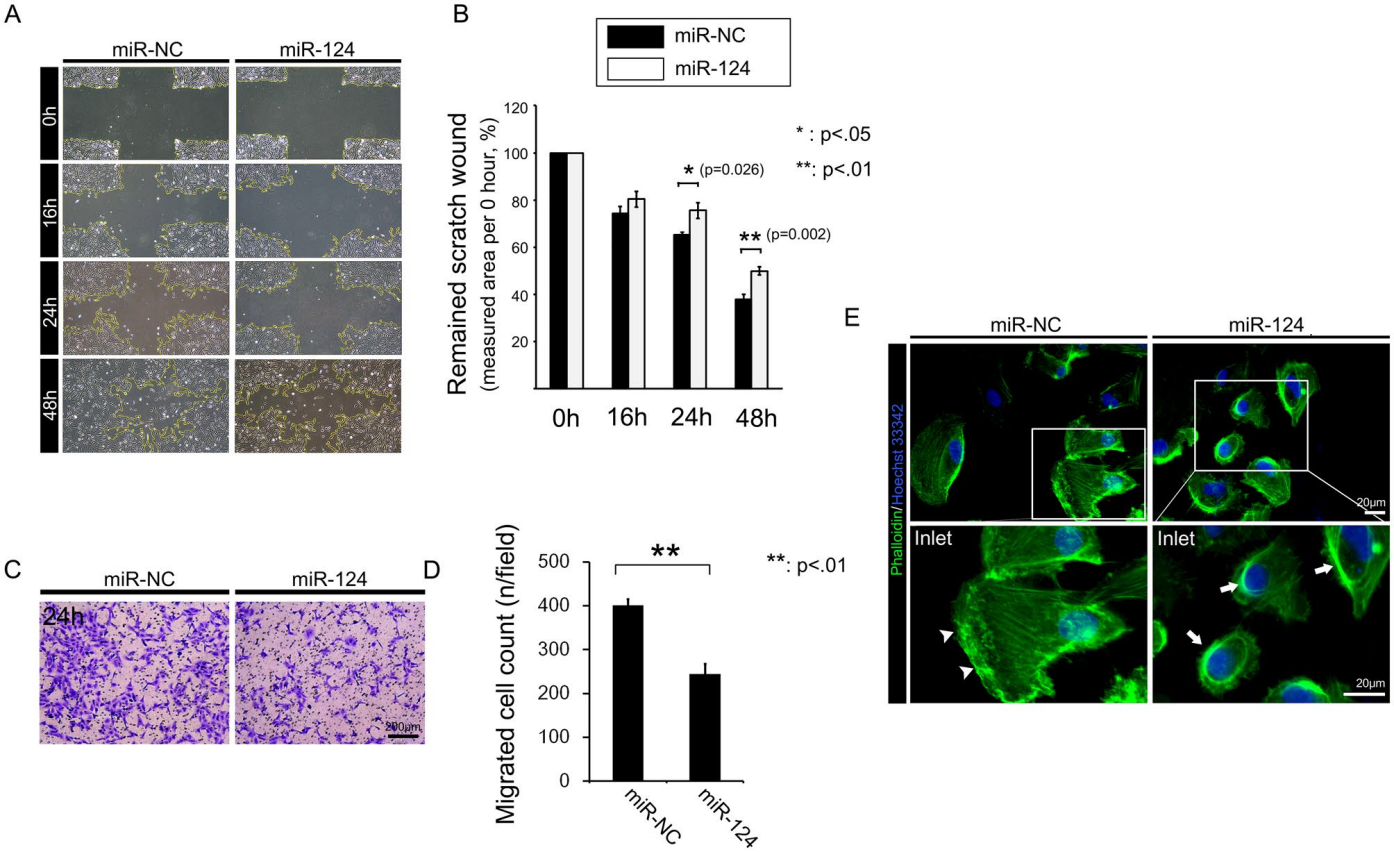
Effects of miR-124 on RPE cell motility. (**A**) Representative images of the cross-hair wound-healing assay. (**B**) Quantitative measurement of the cross-hair wound after cell transfection with miR-NC or miR-124. (**C**) Transwell migration assay for migrating RPE cells 24 h after transfection with miR-124. (**D**) Statistical analysis of transfected versus control migratory RPE cells. (**E**) Filamentous actin staining for lamellipodium formation and cytoplasmic actin condensation. Data represent mean ± SD. Data were analyzed by independent *t* test. *p < 0.05; **p < 0.01.

**Figure 4:**
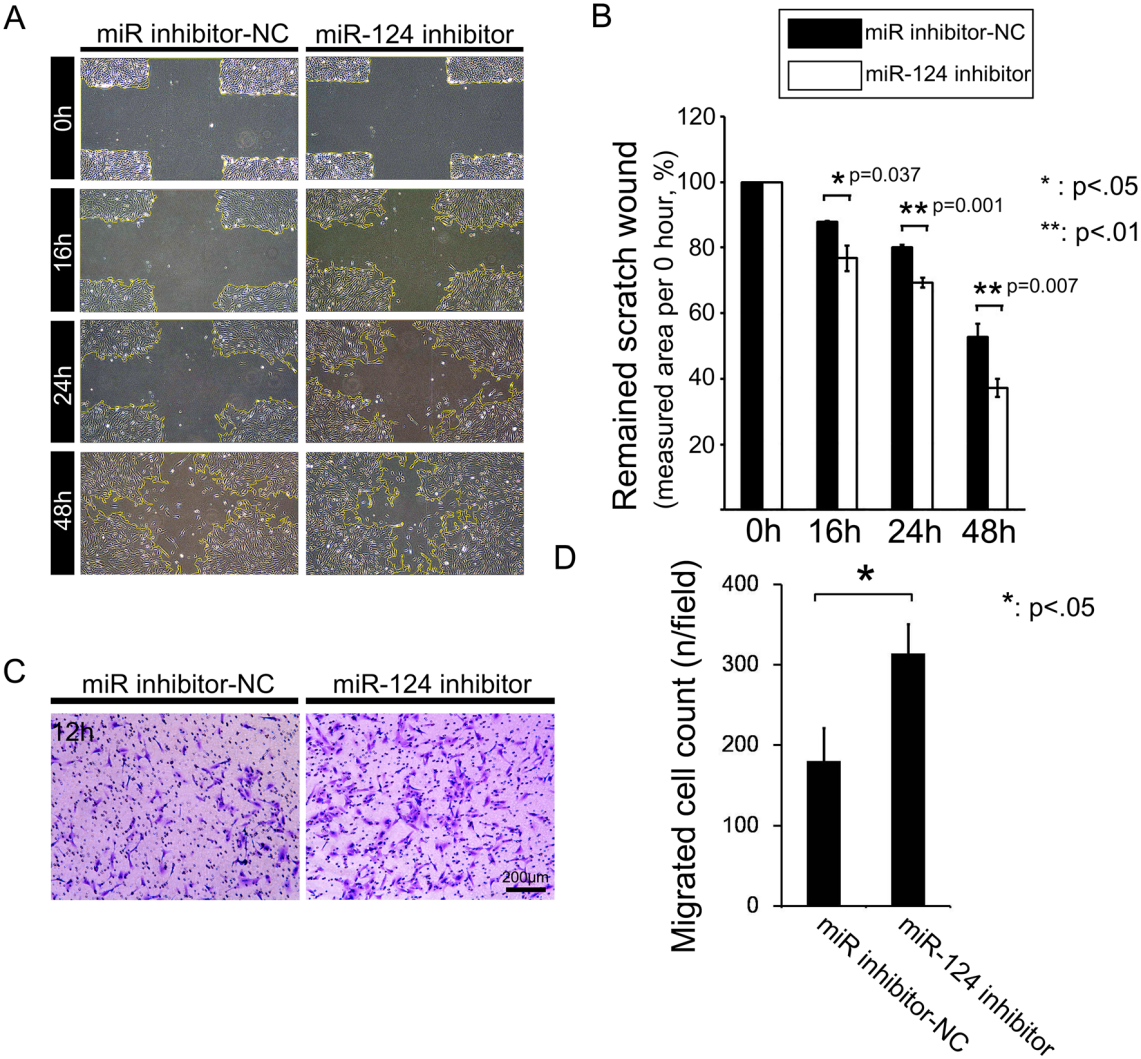
Effects of transfection with an miR-124 inhibitor on RPE cell motility. (**A**) Representative images of the wound closure in monolayers of RPE cells transfected with miR inhibitor-NC and miR-124 inhibitor constructs. (**B**) Statistical analysis of the assay in panel (**A**). (**C**) Representative images from the transwell migration assay with RPE cells transfected with miR inhibitor-NC and miR-124 inhibitor constructs. (**D**) Statistical analysis of the assay in panel (**C**). Data represent mean ± SD. Data were analyzed by independent *t* test. *p < 0.05; **p < 0.01.

We performed in vitro wound-healing and transwell migration assays to examine the effects of miR-124 overexpression on cell motility. Overexpression of miR-124 significantly decreased the wound healing capacity of RPE cells, compared with that of miR-NC treated cells, at 24 and 48 h (Fig. 3A, B). In addition, miR-124 overexpression decreased the number of migratory cells after 12 h (Fig. 3C, D). We investigated the cause of these phenomena by evaluating the cell structure using phalloidin staining. At the leading ends of cell boundaries, miR-124-overexpressing cells lacked lamellipodia, which are crucial for cell motility. In addition, miR-124-transfected cells had altered actin structures, characterised by actin condensation at the cell border (Fig. 3E). Conversely, treatment of RPE cells with the miR-124 inhibitor, designed to antagonizse endogenous miR-124 expression, in RPE cells significantly enhanced wound healing after 16, 24, and 48 h incubations (Fig. 4A, B). In addition, miR-124 inhibitor significantly increased RPE cell migration in the transwell assay, compared with the miR inhibitor-NC (Fig. 4C, D).

### Identification of putative miR-124 target sequences in the ***RHOG*** 3′ UTR

To verify the regulatory effects of miR-124, we investigated potential regulatory targets with an in silico analysis using TargetScan ver. 6.2 (https://www.targetscan.org)^18,19^. *RHOG* (NM_001665) is a well-known regulatory gene related to lamellipodium formation and regulation. A search of the 3′ UTR of human *RHOG* revealed 2 highly evolutionarily conserved seed sequences that are targetable by miR-124 (Supplementary figure S2 A).

### miR-124 directly targeted the ***RHOG*** 3′ UTR in RPE Cells

Based on an in silico analysis, in which *RHOG* 3′ UTR was predicted to be a target of miR-124 in RPE cells, we next performed an *RHOG* 3′ UTR reporter plasmid assay in RPE cells. Co-transfection of the random control reporter plasmid with miR-NC or miR-124 did not affect luciferase activity. On the other hand, co-transfection of the *RHOG* 3′ UTR reporter plasmid with miR-124 reduced the luciferase activity to less than 15% of that observed in control cells co-transfected with miR-NC (Supplementary figure S2 B).

### Knockdown of RHOG negatively regulates cell viability and proliferation

**Figure 5:**
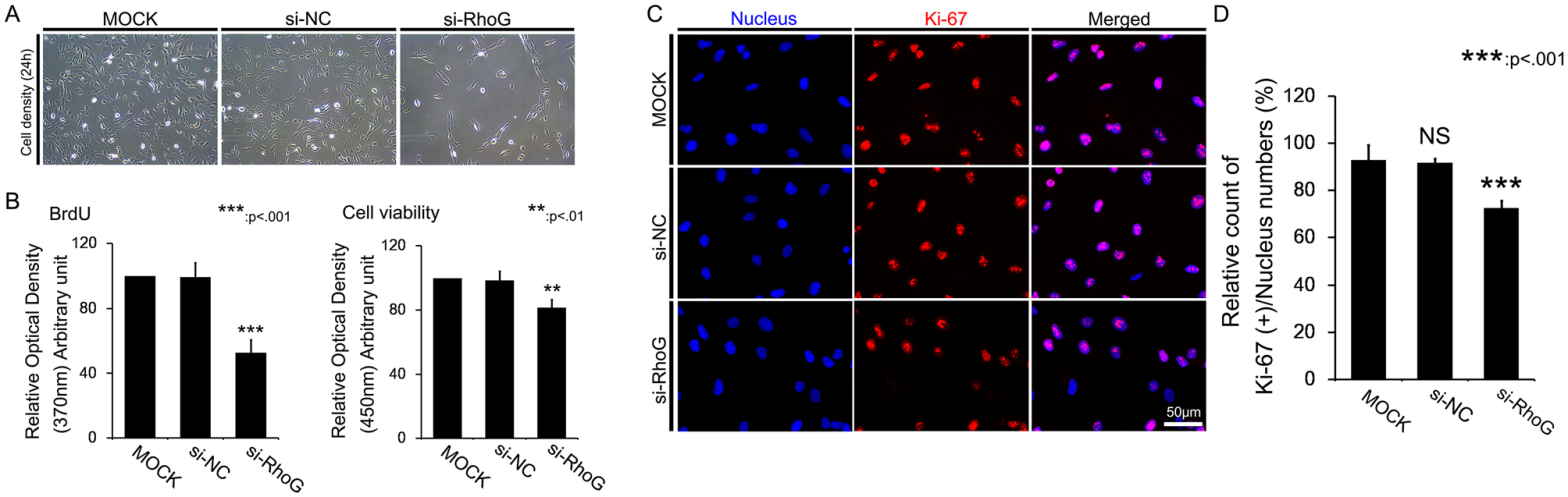
The effects of RhoG knockdown on RPE cell proliferation. (**A**) Representative images of RPE cells transfected with si-RhoG. (**B**) Quantitative analysis of the effects of RhoG knockdown on RPE cell proliferation and viability. (**C**) Immunostaining of Ki-67 and (**D**) statistical analysis of Ki-67 expression after transfection with control or si-RhoG constructs. Data represent mean ± SD. Data were analyzed by one-way ANOVA followed by Turkey’s HSD post hoc test. **p < 0.01; ***p < 0.001.

We evaluated the effects of direct *RHOG* silencing on RPE cell viability and proliferation. Twenty-four hours after transfection of RPE cells with si-*RHOG*, we observed significantly lower cell density in the transfected cells than in the control cultures (Fig. 5A). RHOG-knockdown also reduced cell viability and proliferation (Fig. 5B). In addition, we observed significantly fewer Ki-67-positive cells among the si-*RHOG*-transfected RPE cells than among the controls (Fig. 5C, D).

### Knockdown of RHOG altered RPE cell motility and lamellipodium formation

**Figure 6:**
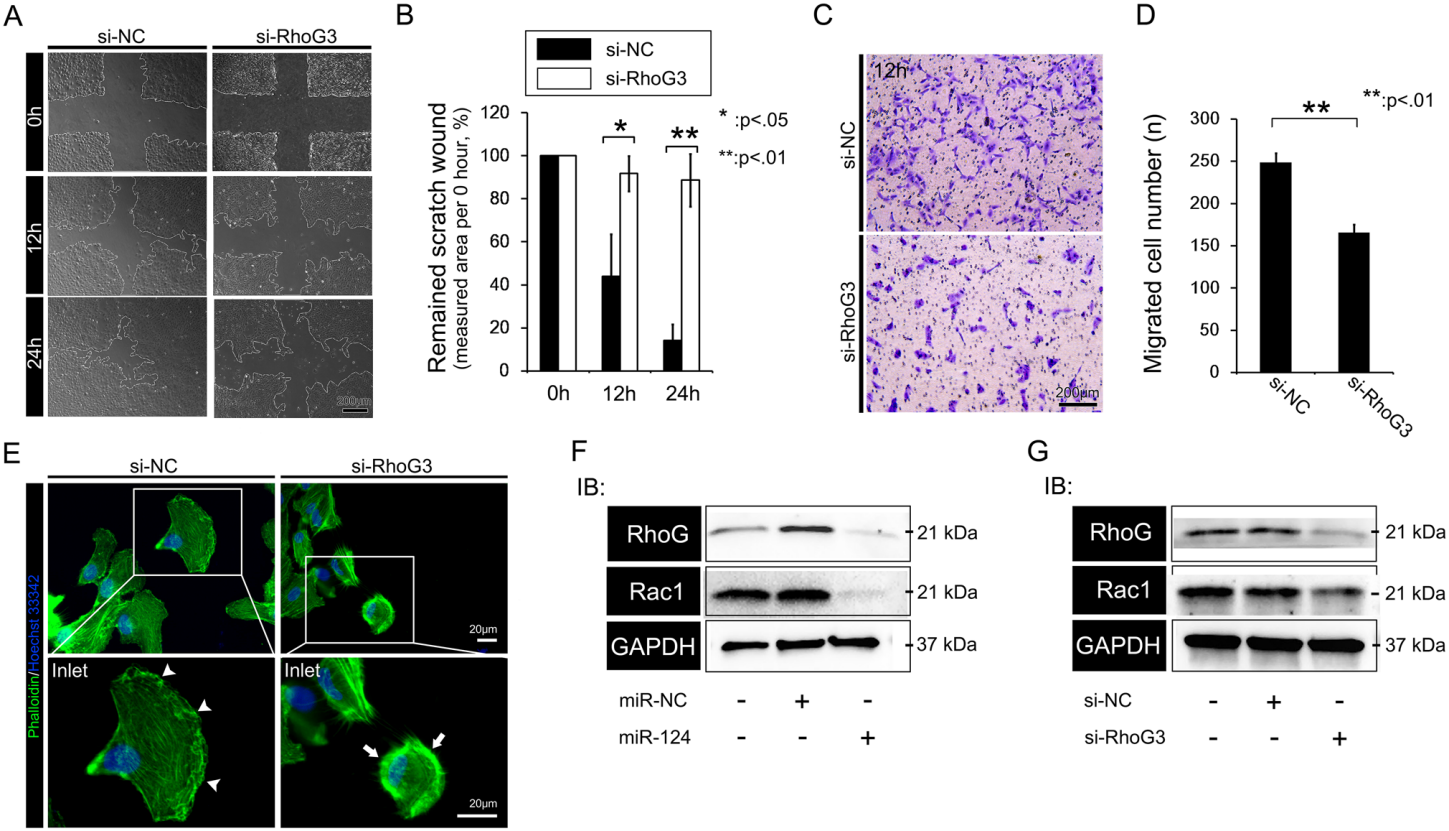
RhoG-silencing effects on RPE migration. (**A**) Representative images of cross-hair wound healing in monolayers of RPE cells transfected with si-RhoG or control constructs. (**B**) Statistical analysis of the assay in panel (**A**). (**C**) Representative images and (**D**) statistical analysis of a transwell migration assay with cells transfected with the control vector or si-RhoG. (**E**) Phalloidin staining for lamellipodium formation and filamentous actin condensation at the borders of RPE cells transfected with control or si-RhoG constructs. (**F**) Western blot analysis of the expression of RhoG and its downstream effector Rac1 in RPE cells transfected with miR-124 and si-RhoG. Data represent mean ± SD. Data were analyzed by independent *t* test. *p < 0.05; **p < 0.01.

We next analyzed the effects of *RHOG* silencing on RPE cell motility and cytoskeletal structure. Monolayers of RPE cells transfected with si-*RHOG* had significantly reduced wound healing ability after cross-hair pattern wounding than the controls (Fig. 6A, B). In addition, transwell migration assays revealed significantly fewer si-*RHOG* transfected migratory cells than migratory control cells (Fig. 6C, D). We also found fewer lamellipodia and less filamentous actin condensation at the borders of RPE cells through phalloidin staining after transfection with si-*RHOG* (Fig. 6E). Western blot analysis after miR-124 knockdown and si-*RHOG* treatment revealed similar knockdowns of RHOG and RAC1 in RPE cells (Fig. 6F).

### Transfection with miR-124 and si-*RHOG* resulted in similar expression patterns of cell cycle regulatory factors

**Figure 7:**
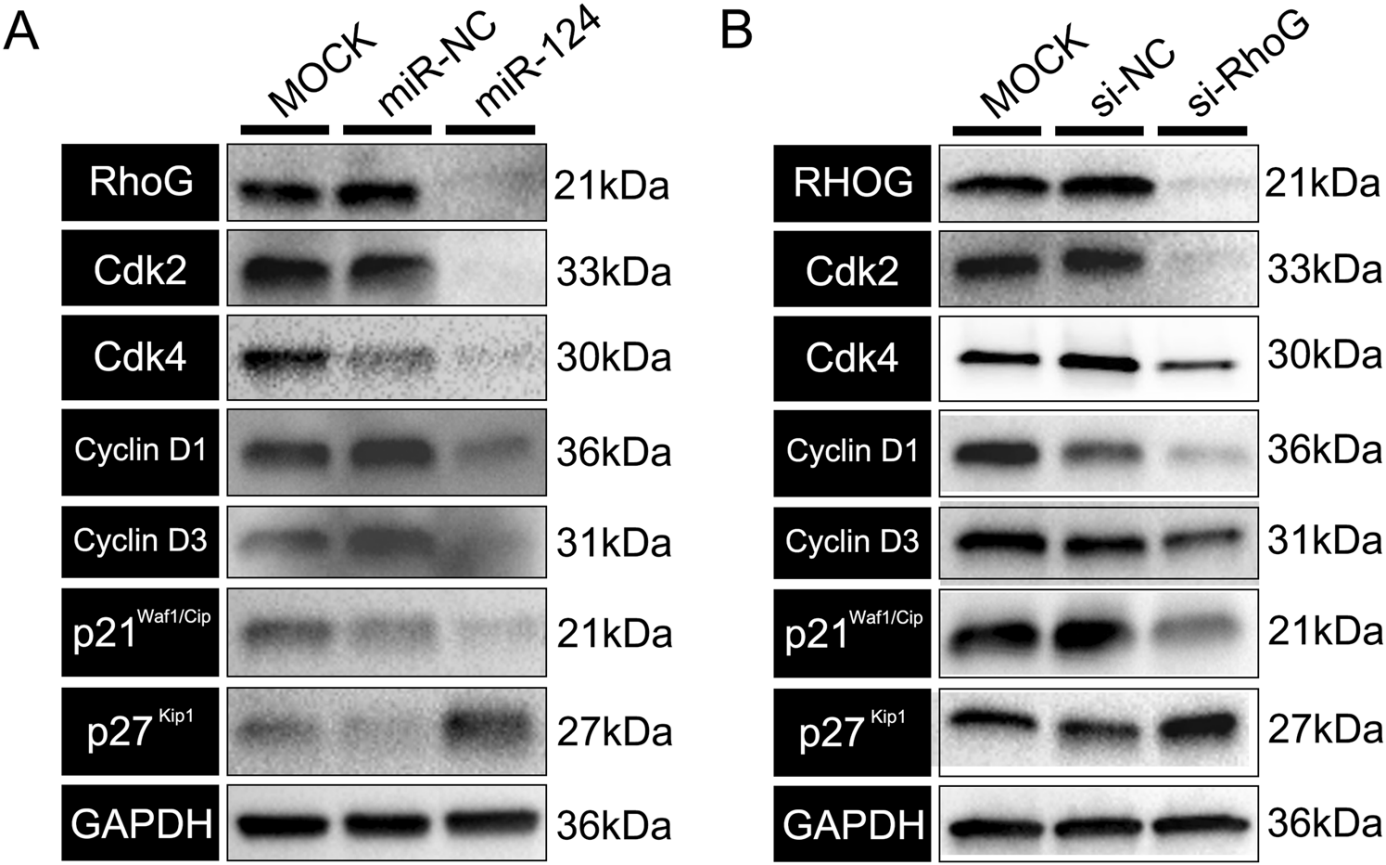
Effects of transfection with miR-124 or si-RhoG on the expression of cell cycle regulation factors in RPE cells. Western blot analysis of Cdk2, Cdk4, Cyclin D1, Cyclin D3, p21^Waf1/Cip1^, and p27^Kip1^ after transfection with (**A**) miR-124 and (**B**) si-RhoG.

Transfection with miR-124 and si-*RHOG* resulted in knockdown of *RHOG* in RPE cells. In addition, miR-124 transfection induced down-regulation of the cell cycle-related factors, Cdk2, Cdk4, p21^Waf1/Cip1^, Cyclin D1, and Cyclin D3, in RPE cells. Conversely, p27^Kip1^ was up-regulated after transfection with miR-124. Knockdown of *RHOG* with siRNA similarly regulated the expression of cell cycle-related proteins (Fig. 7A, B).

### RhoG knockdown or replenishment in the context of miR-124 regulation proved miR-124 plays a pivotal role in RhoG-mediated cell proliferation and migration

**Figure 8:**
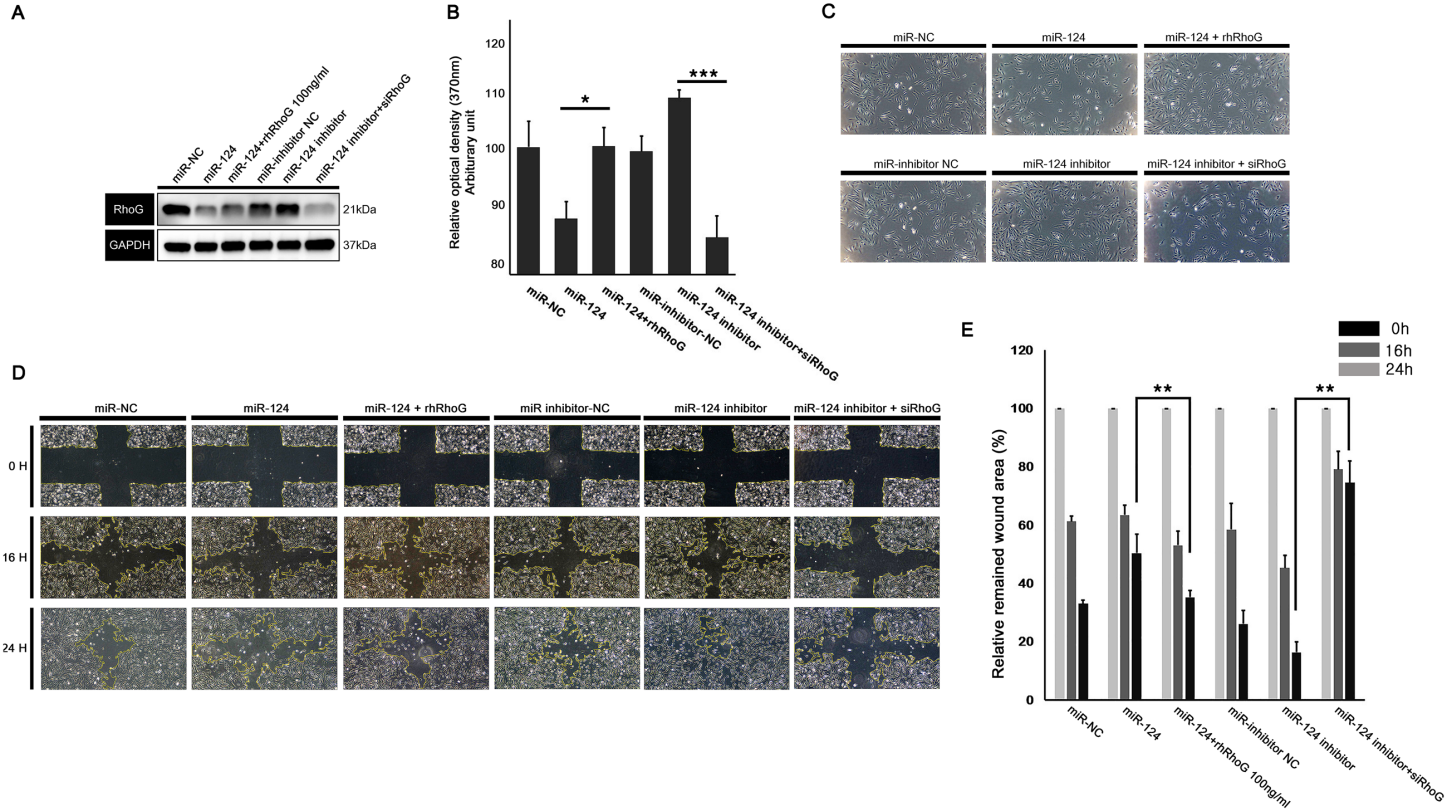
RhoG knockdown and rescue experiments in RPE cells differentially expressing miR-124. (**A**) Western blot analysis of RhoG expression in the context of miR-124 overexpression and RhoG overexpression, or miR-124 inhibition and siRhoG transfection. (**B**,**C**) Quantitative measurements of RPE cell viability (measured via the WST-8 assay) and the respective microscopic images in the context of miR-124 overexpression and RhoG overexpression, or miR-124 inhibition and siRhoG transfection. (**D**,**E**) Wound healing assay and the respective quantitative analysis in the context of miR-124 overexpression and RhoG overexpression, or miR-124 inhibition and siRhoG transfection. Data represent mean ± SD. Data were analyzed by one-way ANOVA followed by Turkey’s HSD post hoc test. *p < 0.05; **p < 0.01; ***p < 0.001.

To definitely disclose the relationship between miR-124 and RhoG, we conducted a series of experiments in the context of simultaneous modulation of miR-124 and RhoG expression. Overexpression of miR-124 together with RhoG replenishment resulted in the rescue of RhoG expression in RPE cells; although RhoG expression was downregulated by miR-124 overexpression, the supplementation with rhRhoG increased the RhoG levels (expectedly), as per the western blot analysis. In addition, RhoG overexpression secondary to miR-124 inhibition was blocked by siRhoG transfection (Fig. 8A). Of note, cell viability and wound healing potential were recovered after treatment with rhRhoG (in the context of miR-124 overexpression), similarly to the observed after inhibition of endogenous miR-124 (Fig. 8B–E).

## Discussion

During development, RPE cells undergo mitotic cell division; however, they become terminally differentiated at the embryonic stage^20^. Hence, human RPE cells remain in a quiescent stage of mitosis in the postnatal period. In an animal model of RPE atrophy, the endothelium rapidly healed via proliferation and migration of neighbouring RPE cells after debridement^21–23^. However, in humans, RPE regeneration is limited and accompanied by pathological epithelial–mesenchymal transition (EMT)^24^. The lack of regenerative capacity in the human CNS, including the retinal system, necessitates studies on cell-based therapeutic modalities for conditions such as dry age-related macular degeneration^25–27^. Contrary to the usual lack of proliferation in the CNS, formation of subretinal membranes in proliferative vitreoretinopathy^11,12^, tractional membranes in diabetic retinopathy^13^, and idiopathic epiretinal membranes^14^ is induced by the abnormal proliferation and migration of RPE cells. Understanding the underlying mechanisms controlling RPE cell proliferation and migration is essential to the development of therapeutic agents for proliferative RPE diseases.

In a previous report, we elucidated the inhibitory effects of miR-124 on the TGF-β1-induced EMT of RPE cells^28^. Although TGF-β1 itself is a potent growth inhibitor of most cell types, we found decreased cell density after transfection of miR-124 into RPE cells^29,30^. Furthermore, RHOG is a known target of miR-124, and its nomenclature emphasizes its role in growth; thus, we hypothesized that miR-124-regulated RhoG would control the cell cycle progression or proliferation of RPE cells^16,28,31^. The expression of miR-124 dynamically changed along with RPE cell confluence, which is consistent with miRNA in situ hybridization findings in murine RPE cells, which revealed decreased expression of miR-124 upon confluence^8^. However, the expression of miR-124 was significantly increased in RPE cells at between 50 and 90% confluence. RhoG expression, which might be affected by increment in endogenous expression of miR-124, showed a similar, but delayed pattern to miR-124 expression. Because miRNAs mainly control the posttranscriptional regulation of their targets, a delayed decrease in RhoG, compared to miR-124, expression is logical. However, although miR-124 was significantly decreased after full confluence, RhoG expression level did not increase again; in fact, it further decreased. This was contrary to our hypothesis that miR-124 regulates RhoG expression. The activity of the Rho protein is determined by a variety of physical factors, such as integrin engagement, confluence, and formation of adherent junctions^32,33^. It was speculated that this pattern would be due to the difficulty to maintain low confluence continuously in an in vitro system; of note, cell-to-cell contact in the context of full confluence during long-term RPE culture has a strong inhibitory effect on cell proliferation^32,33^. Therefore, to demonstrate the regulation of RhoG expression by miR-124, we decided to treat cells with a miR-124 inhibitor for 24 h and compare them with non-treated cells. Importantly, after treatment with the miR-124 inhibitor, RhoG expression was elevated at 72 h compared to that in non-treated cells at the same time-point.

Various studies have reported regulation of RPE cell proliferation by specific miRNAs. Targeting of *PAX6* by miR-328 led to down-regulation of Pax6 and, consequently, increased RPE cell proliferation^34^. Adijanto et al.^35^ also reported that miR-204 and -211 regulate various physiological processes, including RPE cell proliferation. Most recently, miR-34a was reported to regulate RPE cell proliferation by targeting c-Met and LGR4; the authors also reported that proliferating RPE cells had low endogenous expression of miR-34a^10,36^. The proliferation of various tumour cells is controlled by their endogenous expression levels of miR-124. The expression of miR-124 was low in squamous carcinoma and breast cancer cells, and the overexpression of miR-124 suppressed tumour cell proliferation and attenuated viability^37,38^. In those reports, miR-124 targeted SphK1 and Cdk4 in squamous carcinoma and breast cancer cells, respectively. Especially, in the eye, miR-124 is necessary for both cell proliferation and repression of neurogenesis at the optic vesicle stage in *Xenopus*^39^. In brain neural cells, high expression of miR-124 negatively regulates cell cycle progression beyond the G_0_/G_1_ phase by repression of Cdk6^40,41^. We found that Cdk2, Cdk4, Cyclin D1, and Cyclin D3 were down-regulated by miR-124 overexpression, consistent with increased expression of p27^Kip1^, in RPE cells. Since these markers were regulated in the same manner by si-*RHOG* and we could demonstrate that miR-124 mainly targets RHOG (via RhoG knockdown and rescue experiments), we conclude that the miR-124-induced regulation of the proliferation of RPE cells is accomplished via RHOG. Therefore, miR-124 may have an additional mechanism of action in RPE cells compared to that in the neural cells of the CNS. Further research is needed to elucidate the underlying mechanisms driving the increased expression of p27^Kip1^ upon miR-124 overexpression in RPE cells.

Accelerated cell migration, with concurrent cell cycle progression, plays an important role during embryonic development and wound healing. High lens and RPE cell motility contribute to pathological alterations, such as posterior capsule opacification or proliferative vitreoretinopathy-related changes to the retina. The regulation of motility would be a putative therapeutic approach to repress the visual impairment caused by posterior capsule opacification or proliferative vitreoretinopathy. Cell motility is an integrated, multistep cycle, which includes cell polarisation, protrusion, stable focal adhesion, detachment of adhesion, and retraction^42,43^. Small GTPases in the RHO family control elements of cell motility, which led us to hypothesize that the repression of RPE cell motility by miR-124 overexpression acted via the miR-124/RHOG/RAC1 signalling axis. The binding of miR-124 to the 3′ UTR of *RHOG* mRNA, and its regulation of lamellipodium formation, wound healing, and RPE cell migration, are consistent with that hypothesis.

In conclusion, the miR-124/RHOG axis might be a potential therapeutic target in proliferative diseases affecting RPE cells, such as proliferative vitreoretinopathy.

## Methods

### Cell line and cell culture

All experiments in this study used an immortalized human RPE cell line (APRE-19), which was obtained from the American Type Culture Collection (ATCC, Manassas, VA, USA). APRE-19 was maintained in a 1:1 mixture of Dulbecco’s modified Eagle’s medium and Ham’s F12 nutrient supplemented with 10% (v/v) foetal bovine serum (FBS) and 1% (v/v) penicillin/streptomycin in a humidified incubator at 37 °C with 5% CO_2_ (all reagents from Welgene, Daegu, Korea). The cell line was used in experiments between passages 10 and 18.

### RNA interference constructs and reagents

mirVana™ miR-124 mimic (miR-124), miRNA mimic negative control (miR-NC), mirVana™ miR-124 inhibitor, miRNA inhibitor-NC, Lipofectamine® RNAiMAX and Lipofectamine™ 3,000 transfection reagent, and Alexa Fluor® 488-conjugated phalloidin were purchased from ThermoFisher Scientific (Waltham, MA, USA). The siRNA NC and 4 predesigned *RHOG* siRNAs (si-*RHOG*) with different sequences were from Qiagen (Hilden, Germany). Vectashield® Mounting Medium with DAPI was purchased from Vector Laboratories (Peterborough, UK). The 5-bromo-2′-deoxyuridine (BrdU) enzyme-linked immunosorbent assay (ELISA) kit was purchased from Roche Diagnostics (Basel, Switzerland), Cell Counting Kit-8 (CCK-8) from Dojindo Molecular Technologies (Rockville, MD, USA), and the transwell migration kit from Cell Biolabs (San Diego, CA, USA). The following primary antibodies were used: anti-RHOG from Sigma-Aldrich (St. Louis, MO, USA); anti-RAC1 from EMD Millipore (Billerica, MA, USA); anti-GAPDH, Cyclin D1, D3, Cdk2, Cdk4, p21^Waf1/Cip1^, and p27^Kip1^ from Cell Signaling Technology (Danvers, MA, USA); and anti-Ki-67 from Merck Millipore (Billerica, MA, USA) and Abcam (Cambridge, UK). All secondary antibodies for immunoblot assays were from Santa Cruz Biotechnology (Santa Cruz, CA, USA) and Jackson Laboratory (Bar Harbor, ME, USA), and fluorescently conjugated secondary antibodies for immunostaining were from Molecular Probes (Eugene, OR, USA). All other chemicals were obtained from Sigma-Aldrich (St. Louis, MO, USA).

### Quantitative analysis of relative miR-124 expression

To investigate the relative expression levels of endogenous miR-124 depending on RPE cell density, ARPE-19 cells were plated at a density of 7.5 × 10^4^ cells/mL in 6-well cell culture plates. At 24, 48, 72, and 96 h after plating, miRNA was extracted and miRNA real-time quantitative PCR (qPCR) analysis was performed. At each time point, proteins were also extracted for the detection of relative RHOG expression. The culture medium was aspirated and cells were rinsed thrice with phosphate-buffered saline (PBS); thereafter, total RNA was extracted using QIAzol lysis reagent (Qiagen, Hilden, Germany). A 1-μg aliquot of total RNA was reverse transcribed using miScript II Reverse Transcriptase (Qiagen, Hilden, Germany). qPCR was performed using QuantiTect® SYBR® Green PCR Master Mix and the Bio-Rad CFX96 Real-Time PCR Detection System (Bio-Rad Laboratories, Hercules, CA, USA). The expression of miR-124 was normalised to that of the control *RNU6* RNA or miR-16 using the 2^−ΔΔCT^ method^17^.

### Cell viability and proliferation assays

ARPE-19 cells were plated in 96-well plates and subsequently transfected with oligonucleotides (miRNA mimic or siRNA) after 24 h. We introduced 50 nM miR-NC or miR-124 into the cells with RNAiMAX transfection reagent over 6 h, and 50 nM of si-NC or si-*RHOG* molecules were transfected using the same reagent. Twenty-four hours after transfection, cell viability or BrdU incorporation assays were performed. Cell viability was evaluated with a CCK-8 assay. Briefly, the culture medium was aspirated and cells were rinsed thrice with PBS. A mixture of 10 µL reagent with 90 µL serum-free medium was added to each well of a 96-well plate. After a 4-h incubation, colorimetric detection was performed at 450 nm with a microplate reader (VersaMax, Molecular Devices, Sunnyvale, CA, USA). Proliferation was assessed with a BrdU ELISA kit. Briefly, BrdU labelling reagent was added to the culture medium and incubated for 2 h prior to the assay. Culture medium was aspirated after 2 h, followed by fixation, denaturation, and anti-BrdU peroxidase conjugation. The substrates were added to each well and colorimetric detection was performed with a microplate reader. All experiments were performed in triplicate.

### Western immunoblot analyses

Cells were rinsed thrice with cold PBS and lysed in radioimmunoprecipitate buffer. The protein concentrations of the lysates were measured using a protein bicinchoninic acid assay kit (ThermoFisher Scientific, Waltham, MA, USA). Equal quantities of total protein samples were loaded in 8–15% polyacrylamide gels for sodium dodecyl sulphate polyacrylamide gel electrophoresis. The resolved proteins were electroblotted onto nitrocellulose membranes and blocked with 5% skim milk solution for 1 h. After overnight incubation with the appropriate primary antibodies, the membranes were incubated with secondary antibodies for 1 h. GAPDH was used as a loading control for blot band densitometry.

### Phalloidin staining and immunocytochemistry

ARPE-19 cells were plated on 4-well cell culture slides (SPL Life Sciences, Pocheon, Korea). The miRNA or siRNA constructs were delivered 24 h after plating and the cells were cultured to 50–60% confluency. The cells were rinsed thrice with PBS and fixed with 4% paraformaldehyde for 30 min. For observation of filamentous actin, the cells were stained with Alexa Fluor® 488-conjugated phalloidin and Hoechst 33,342 solution. For immunocytochemical analyses, cells were permeabilised with 0.4% (v/v) Triton™ X-100 for 15 min and blocked with 1% (w/v) bovine serum albumin in 0.1% (v/v) Tween® 20 in PBS for 30 min. They were incubated with anti-Ki-67 primary antibodies for 12 h, then Alexa Fluor® 594-conjugated secondary antibodies (Molecular Probes, Eugene, OR, USA) for 2 h at room temperature. RPE cell nuclei were stained with Vectashield® mounting solution containing 4′,6-diamidino-2-phenylindole (DAPI) after several rinses. Each slide was observed using an upright fluorescent microscope (Axio Imager 2; Zeiss, Cologne, Germany) and images were obtained using AxioVision SE64 version 4.9.1.0 software (Zeiss, Cologne, Germany). Ki-67-positive cells were counted at 5 different locations, and the percentage of Ki-67-positive cells of DAPI-positive cells was calculated. All experiments were performed in triplicate.

### Wound-healing assay

APRE-19 cells were plated into 12-well plates and allowed to reach 100% confluence. After cell transfection with RNA interference constructs (miRNA mimic, miRNA inhibitor, or siRNA), a cross-hair linear wound was inflicted with a 200-µL pipette tip followed by treatment with 50 μg/ml mitomycin C for 1 h. After 3 PBS rinses, medium containing 1% FBS was added. The remaining wound was observed at each indicated time point and measured under phase-contrast microscopy using ImageJ (https://imagej.nih.gov/ij/; provided in the public domain by the National Institutes of Health, Bethesda, MD, USA). All experiments were performed in triplicate.

### Transwell migration assay

After transfection with miRNA or siRNA, cells were trypsinised and resuspended in serum-free medium. Medium containing 10% FBS was added to the bottom chambers of the wells and cell suspensions were poured into polycarbonate membrane inserts with 8-µm pores. After a 12-h incubation, the cells that had migrated to the bottom of the polycarbonate membrane were fixed with 4% paraformaldehyde and stained with haematoxylin. Migratory cells were counted with a phase-contrast microscope at 3 different locations. All experiments were performed in triplicate.

### Plasmid transfection and luciferase reporter assay

A random control luciferase plasmid and LightSwitch™ *RHOG* mRNA 3′ untranslated region (UTR) plasmid were purchased from SwitchGear Genomics (Menlo Park, CA, USA). Cells were plated in 96-well luminescence plates and conventional 96-well cell culture plates prior to transfection and cultured up to 70% confluence over 24 h. The cells were co-transfected for 6 h, with either random control or *RHOG* mRNA 3′ UTR luciferase plasmid and either miR-124 or miR-NC, using Lipofectamine™ 3,000 reagents, then incubated for 24 h. The transfected cells were lysed with buffer-substrate solution. After 30 min of incubation, luciferase activity of each well was detected with a luminometer (Centro LB 960; Berthold Technologies, Bad Wildbad, Germany). All experiments were performed in triplicate.

### Statistical analysis

Representative data obtained from experiments conducted in triplicate are presented as mean ± standard deviation. Data were evaluated using independent *t* tests, or one-way analysis of variance (ANOVA) and Tukey’s honest significant difference post hoc test, to identify statistically significant differences. A *p* value < 0.05 was regarded as statistically significant.

## Supplementary information

Supplementary Information.
