# Supplementary material for: Regulation of Ras homolog family member G by microRNA-124 regulates proliferation and migration of human retinal pigment epithelial cells
Source: Sci Rep. 2020 Sep 22;10:15420. doi: 10.1038/s41598-020-72360-5 (PMC7508981; doi:10.1038/s41598-020-72360-5)
Supplement: Supplementary file 1 — Supplementary Information. [file 41598_2020_72360_MOESM1_ESM.pdf]

Regulation of Ras homolog family member G by microRNA-124 regulates proliferation and migration of human retinal pigment epithelial cells

Jong Hwa Jun[1*], Myeong-Jin Son[1], Hyun-Gyo Lee[1], Kyu Young Shim[1], Won-Ki Baek[2], Jae-Young Kim[3], and Choun-Ki Joo[4]

Regulation of Ras homolog family member G by microRNA-124 regulates proliferation and migration of human retinal pigment epithelial cells

Jong Hwa Jun[1*], Myeong-Jin Son[1], Hyun-Gyo Lee[1], Kyu Young Shim[1], Won-Ki Baek[2], Jae-Young Kim[3], and Choun-Ki Joo[4]

[1]Department of Ophthalmology, Keimyung University School of Medicine, Dongsan Medical Center, Daegu, Korea

[2]Department of Microbiology, Keimyung University School of Medicine, Daegu, Korea

[3]Department of Oral Biochemistry, School of Dentistry, IHBR, Kyungpook National University, Daegu, Korea

[4]Department of Ophthalmology and Visual Science, Seoul St. Mary's Hospital, College of Medicine, The Catholic University of Korea, Seoul, Korea

*Corresponding author

E-mail: junjonghwa@gmail.com

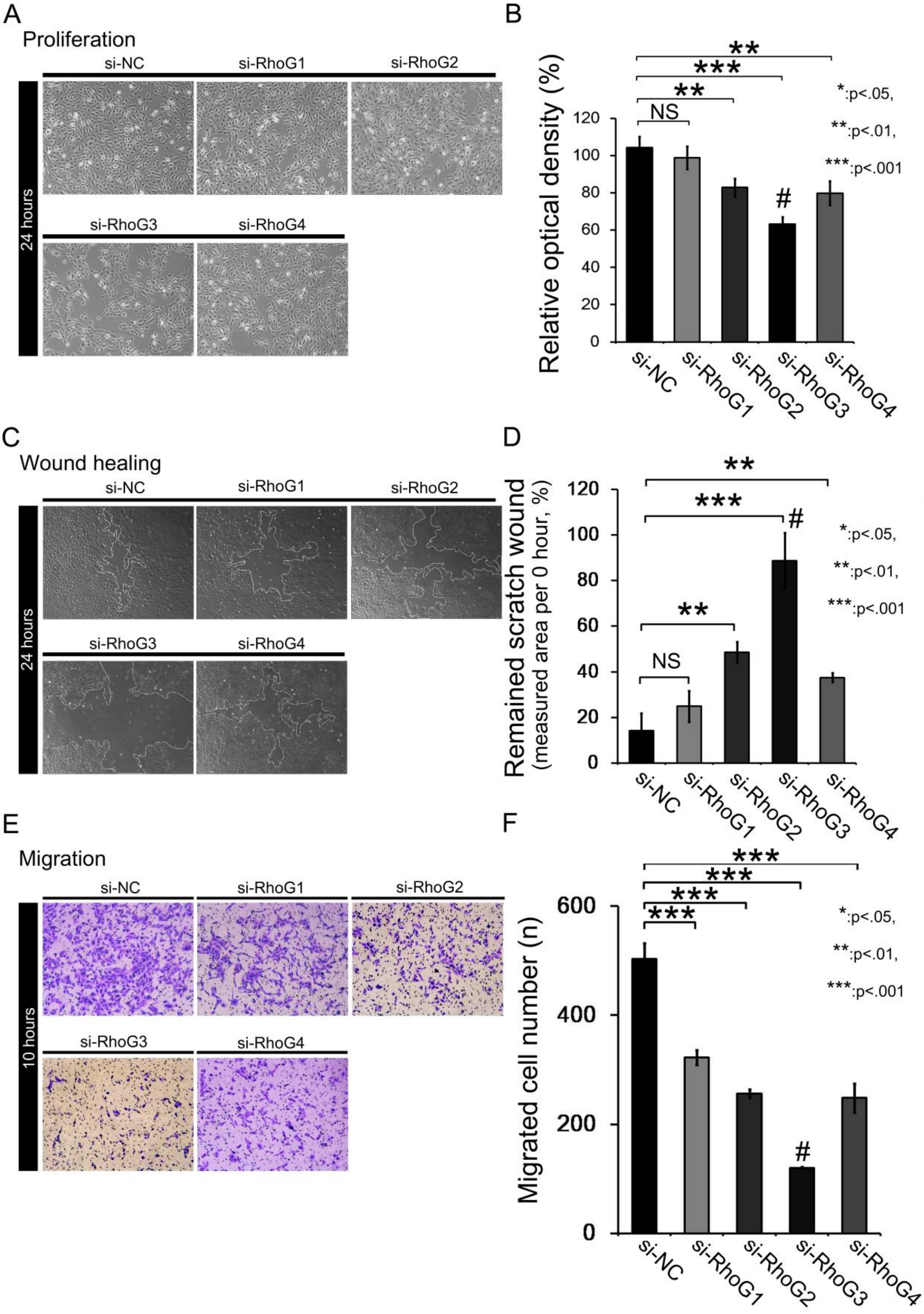
A
Proliferation
si-NC
si-RhoG1
si-RhoG2
si-RhoG3
si-RhoG4
24 hours
B
Relative optical density (%)
0
20
40
60
80
100
120
NS
**
***
**
#
*:p<.05,
**:p<.01,
***:p<.001
si-NC
si-RhoG1
si-RhoG2
si-RhoG3
si-RhoG4
C
Wound healing
si-NC
si-RhoG1
si-RhoG2
si-RhoG3
si-RhoG4
24 hours
D
Remained scratch wound
(measured area per 0 hour, %)
0
20
40
60
80
100
120
NS
**
***
**
#
*:p<.05,
**:p<.01,
***:p<.001
si-NC
si-RhoG1
si-RhoG2
si-RhoG3
si-RhoG4
E
Migration
si-NC
si-RhoG1
si-RhoG2
si-RhoG3
si-RhoG4
10 hours
F
Migrated cell number (n)
0
200
400
600
***
***
***
***
#
*:p<.05,
**:p<.01,
***:p<.001
si-NC
si-RhoG1
si-RhoG2
si-RhoG3
si-RhoG4

Supplementary figure S1. Comparative analysis of 4 si-RhoG sequences on the proliferation, wound healing, and migration of RPE cells. (A) Representative images of RPE cell density after transfection with 4 types of si-RhoG. (B) Statistical analysis of cell viability after transfection with the 4 si-RhoG constructs. (C) Wound healing assay of RPE cells after transfection with the 4 si-RhoG constructs. (D) Statistical analysis of the remaining wound area 24 h after wounding. (E) Representative images of the transwell migration assay with RPE cells transfected with the 4 types of si-RhoG. (F) Statistical analysis of the transwell migration assay in (E). Data represent mean ± SD. Data were analyzed by one-way ANOVA followed by Turkey's HSD test. *, $p < 0.05$; **, $p < 0.01$; ***, $p < 0.001$.

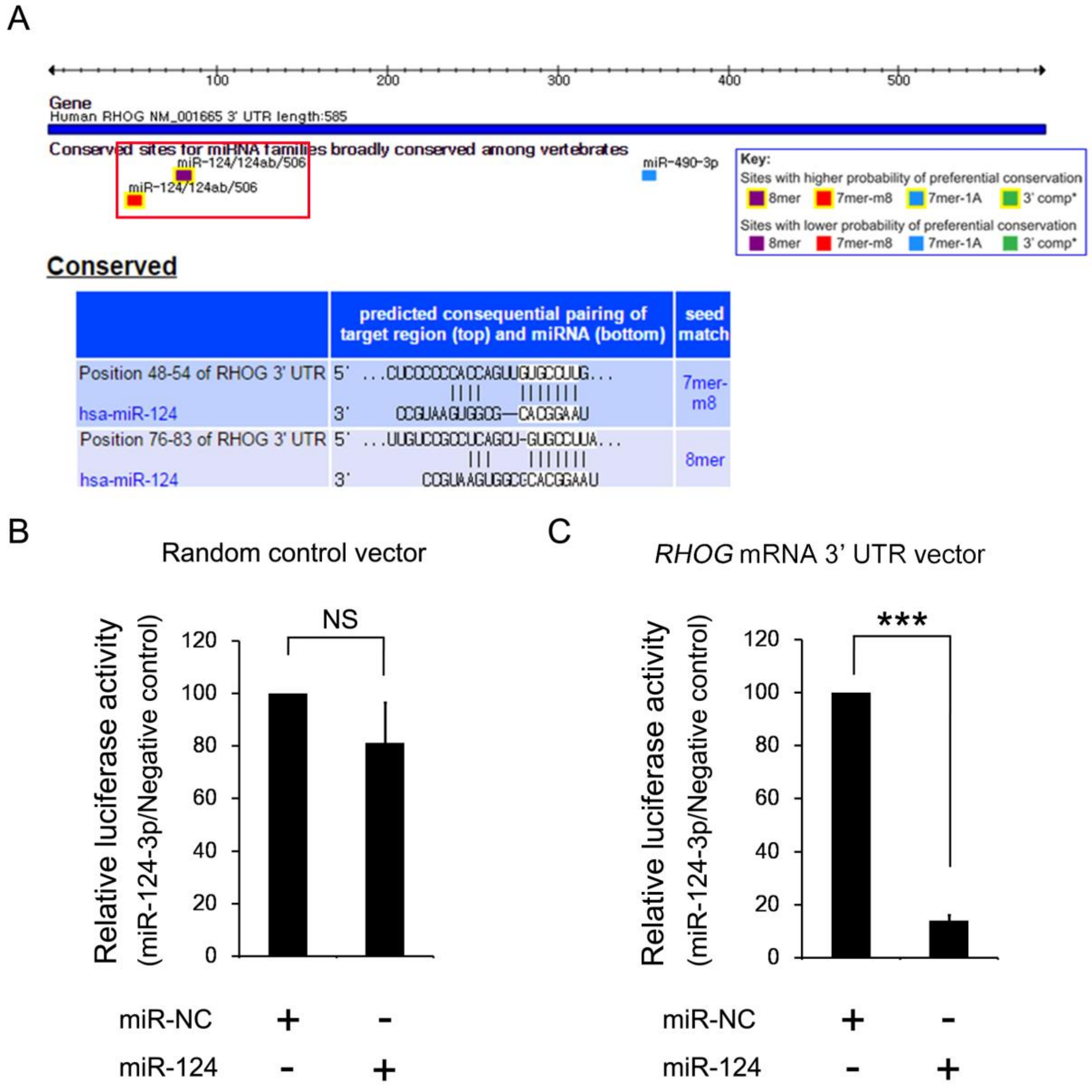

Supplementary figure S2. Analysis of miR-124 binding to the *RHOG* mRNA 3´ UTR. (A) *In silico* assessment of conserved miR-124 binding sequences in the 3´ UTR of *RHOG* mRNA. (B) Luciferase assay for binding to a random control vector in cells transfected with miR-NC or miR-124. (B) Luciferase assay for binding to the *RHOG* mRNA 3´ UTR vector in cells transfected with miR-NC or miR-124. Data represent mean ± SD. Data were analyzed by independent t-test. NS, $p > 0.05$; ***, $p < 0.001$.

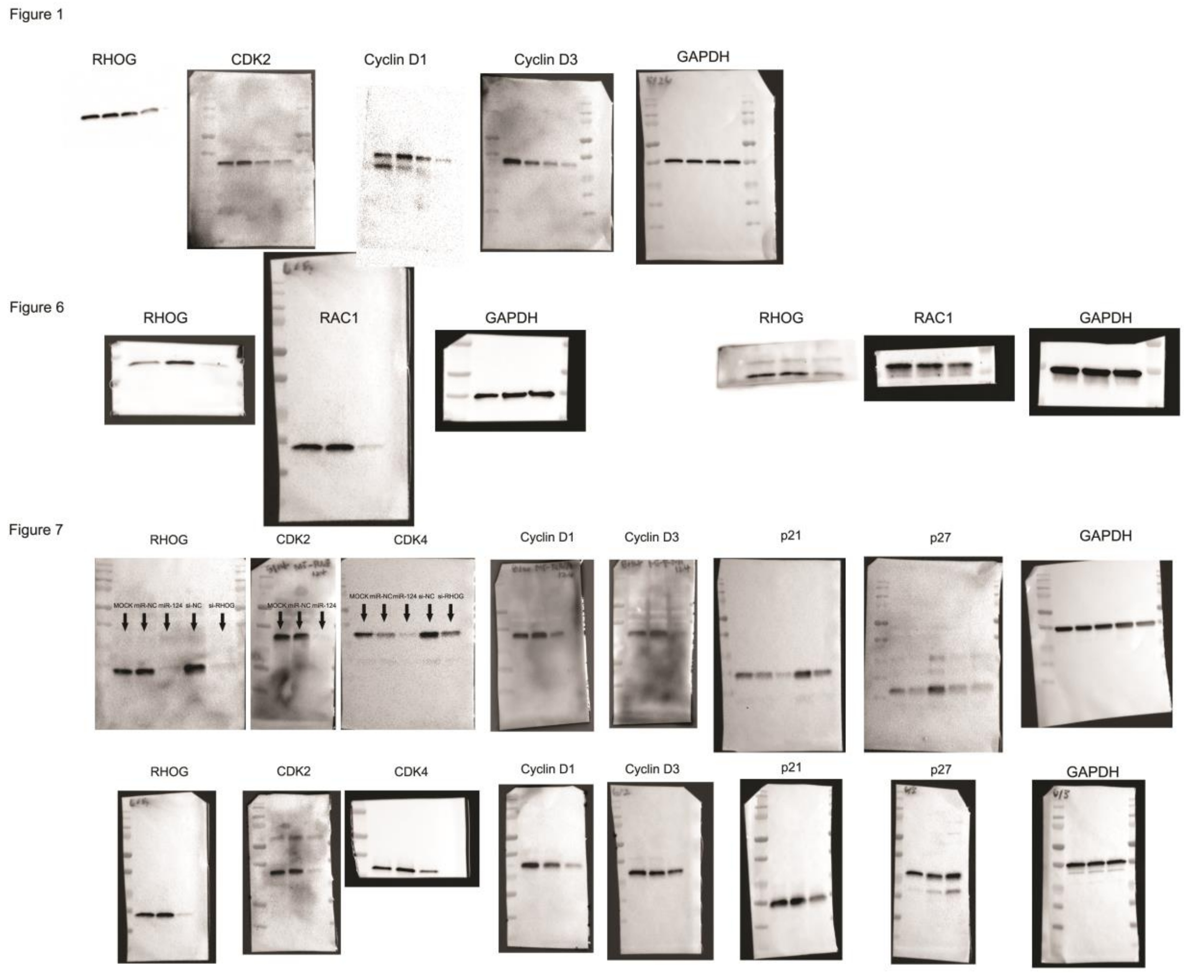

Supplementary figure S3. Uncropped western blot images.
